# Further Quinolizidine Derivatives as Antiarrhythmic Agents- 3

**DOI:** 10.3390/molecules28196916

**Published:** 2023-10-03

**Authors:** Bruno Tasso, Laura Beatrice Mattioli, Michele Tonelli, Vito Boido, Alberto Chiarini, Fabio Sparatore, Roberta Budriesi

**Affiliations:** 1 Department of Pharmacy, University of Genova, 16132 Genova, Italy; bruno.tasso@unige.it (B.T.); michele.tonelli@unige.it (M.T.); vito.boido@gmail.com (V.B.); 2 Food Chemistry and Nutraceutical Research Unit, Department of Pharmacy & Biotechnology, Alma Mater Studiorum, University of Bologna, 40126 Bologna, Italy; laura.mattioli13@unibo.it (L.B.M.); alberto.chiarini@unibo.it (A.C.); roberta.budriesi@unibo.it (R.B.)

**Keywords:** quinolizidine derivatives, lupinine, cytisine, sparteine, antiarrhythmic activity, inotropic and chronotropic activities, vasorelaxant activity

## Abstract

Fourteen quinolizidine derivatives, structurally related to the alkaloids lupinine and cytisine and previously studied for other pharmacological purposes, were presently tested for antiarrhythmic, and other cardiovascular effects on isolated guinea pig heart tissues in comparison to well-established reference drugs. According to their structures, the tested compounds are assembled into three subsets: (a) N-(quinolizidinyl-alkyl)-benzamides; (b) 2-(benzotriazol-2-yl)methyl-1-(quinolizidinyl)alkyl-benzimidazoles; (c) N-substituted cytisines. All compounds but two displayed antiarrhythmic activity that was potent for compounds **4**, **1**, **6**, and **5** (in ascending order). The last compound (N-(3,4,5-trimethoxybenzoyl)aminohomolupinane) was outstanding, exhibiting a nanomolar potency (EC_50_ = 0.017 µM) for the increase in the threshold of ac-arrhythmia. The tested compounds shared strong negative inotropic activity; however, this does not compromise the value of their antiarrhythmic action. On the other hand, only moderate or modest negative chronotropic and vasorelaxant activities were commonly observed. Compound **5**, which has high antiarrhythmic potency, a favorable cardiovascular profile, and is devoid of antihypertensive activity in spontaneously hypertensive rats, represents a lead worthy of further investigation.

## 1. Introduction

Many important therapeutic agents are of natural origin, mainly of alkaloidal nature. However, many other alkaloids, even those exhibiting potent and interesting pharmacological activities, do not find therapeutic application because of their associated negative characteristics. Among these, many are still worthy of consideration, since, as a consequence of their chemical structure, they are susceptible to modifications that are able to enhance their potency and/or induce a upsurge of novel activities unrelated to the original ones.

From this perspective, particular relevance has been attributed to the quinolizidine alkaloids (lupinine, cytisine, sparteine, lupanine, matrine, etc.), which are present in many species of Fabaceae (Lupinus, Genista, Cytisus, Sophora, etc.) [1]. Moreover, the quinolizidine bicycle is also embodied in the structure of alkaloids that not biogenetically related to the cited lupine alkaloids, such as reserpine, vincamine, emetine, and others, but endowed with a variety of biological properties.

We have long been engaged in structural modifications of lupine alkaloids, mainly lupinine and cytisine, in order to exploit these natural resources in the biomedical area. Interesting results were achieved in several pharmacological fields, particularly as antiarrhythmic agents [2,3,4,5].

Arrhythmia is a complex abnormality of cardiac rhythm, affecting an increasing per cent of the population with the increase in age. Arrhythmias are commonly induced or accompanied by heart disease but may occur in patients with other diseases and under certain drug therapies, and are, in themselves, a danger to the patients. Atrial fibrillation is the most common cardiac arrhythmia, often associated with thrombo-embolic stroke.

At present, there are many drugs tht can suppress dysrhythmic cardiac activity through different mechanisms, as illustrated in the classification of Vaughan [6,7], recently updated by M. Lei et al. [8]. However, a satisfactory pharmacological therapy has not yet been developed, and the search for new antiarrhythmic agents is still ongoing, particularly with the aim of obtaining compounds with multiple mechanisms of action, in order to counterbalance the pro-arrhythmic risk inherent to some otherwise valuable types of drug (such as those of class III) [9].

A characteristic of our previously described antiarrhythmic agents [2,3,4,5] is the presence, in their structures, of the bulky, highly lipophilic, and strongly basic quinolizidine moiety present in bi-, tri-, and tetracyclic lupine alkaloids. 

Indeed, this structural feature is embodied in the molecule of sparteine, which was largely used [10] to slow the heart in tachycardia of various origins, and which has been reappraised as an interesting antiarrhythmic agent, as in [11], or in the form of C-substituted derivatives [12,13,14,15], or truncated molecular analogs [16,17,18,19,20,21,22,23]. More recently, the tetracyclic alkaloids matrine and oxymatrine have shown interesting antiarrhythmic properties, which are less potent than quinidine [24,25].

**Figure 1:**
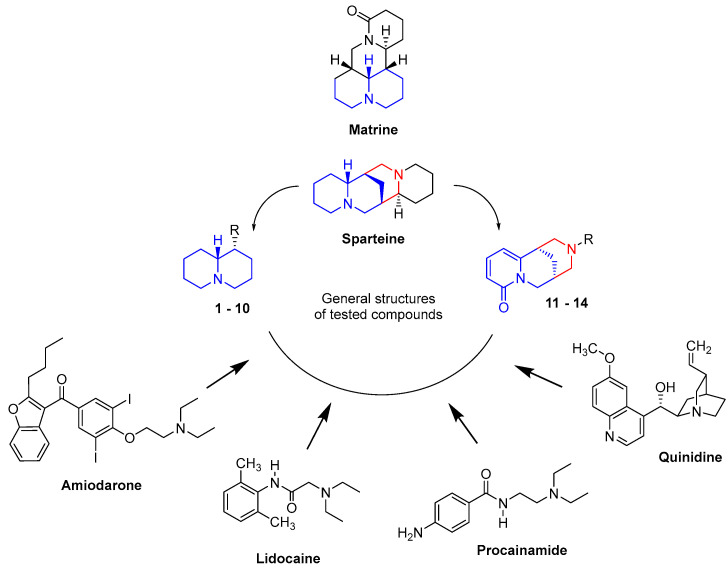
Drug design of tested compounds.

Our approach was (and is) to hybridize a truncated portion of the sparteine molecule (as a quinolizidinylmethyl-residue) with the aromatic moieties present in other well-established antiarrhythmic drugs, such as procainamide, lidocaine, amiodarone, and quinidine (Figure 1).

The previously investigated quinolizidinyl derivative exhibited remarkable antiarrhythmic activity in mice subjected to deep chloroform anesthesia or aconitine infusion [2,3], or electrically driven isolated guinea pig (gp) left atria [4,5], and was often more active and potent than amiodarone, lidocaine, procainamide and quinidine.

**Figure 2:**
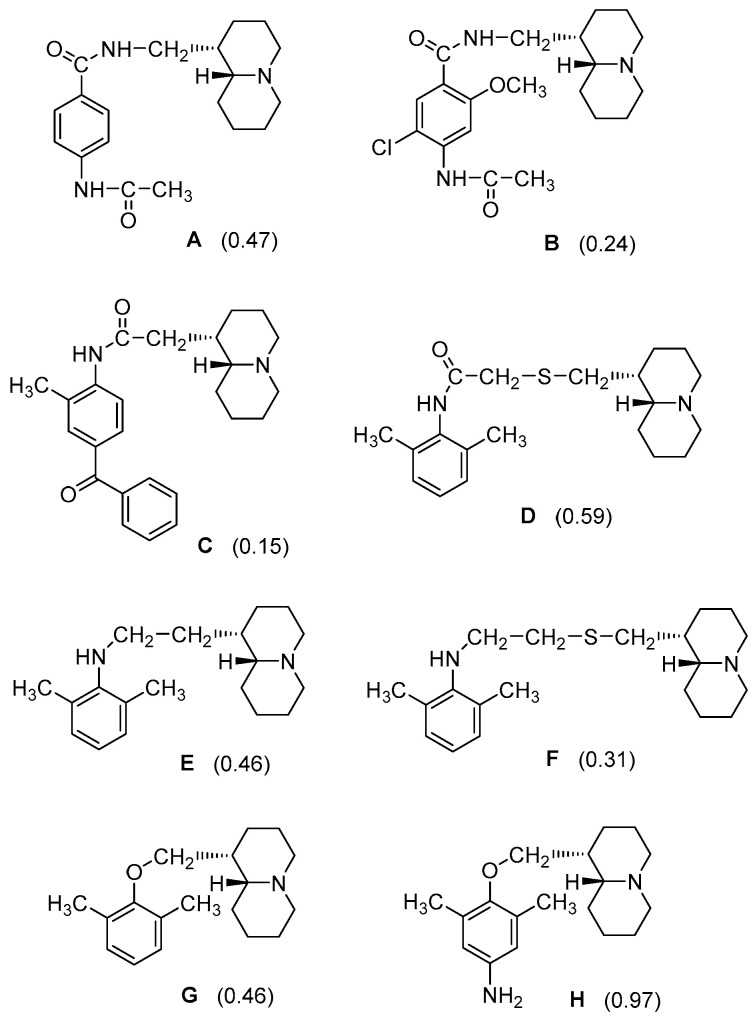
Structures (**A**–**H**) of the most potent previously studied anti-arrhythmic quinolizidine derivatives for (**A**,**G**,**H**) [4]; for (**B**–**F**) [5]; (EC_50_: μM).

In particular, in the in vitro test, many compounds exhibited an EC_50_ even lower than 1 µM (Figure 2), while for quinidine, the most potent of the reference drugs, EC_50_ = 10.26 µM. When additionally tested for inotropic, chronotropic and calcium antagonist activity on isolated gp heart tissues, these compounds displayed an interesting profile, competing favorably with reference compounds.

**Figure 3:**
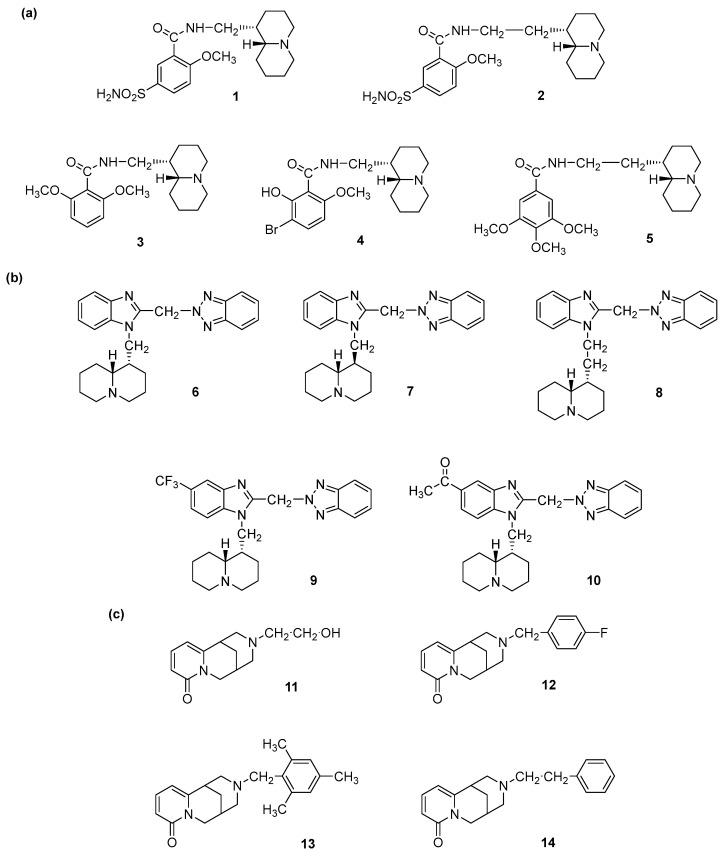
Structures of the presently investigated quinolizidine derivatives (**1**–**14**).

Therefore, we deemed it interesting to pursue the investigations of the cardio-vascular profile of additional quinolizidine derivatives that were available in our in-house library, and could be allocated to three structural subsets (Figure 3):

(a) *N-(Quinolizidinyl-alkyl)-benzamides* (**1**–**5**), related to the previously studied compounds **A** and **B;**
(b) *1-(Quinolizidinyl)alkyl-2-(benzotriazol-2-yl)methyl benzimidazoles* (**6**–**10**), in which the N-(quinolizidinyl)alkylaniline substructure of compounds **E** and **F** is embodied. These compounds, together with several analogs, were previously studied by us as analgetics [26,27] and antivirals [28].
(c) *N-Substituted cytisines* (**11**–**14**), characterized by the presence of three of the four rings of sparteine’s molecular scaffold. The investigated compounds were selected from a large number of cytisine derivatives, previously studied by us as ligands for a neuronal nicotine receptor [29].

## 2. Results and Discussion

Compounds **1**–**14** (Figure 3), together with amiodarone, lidocaine, procainamide, and quinidine as reference drugs, were evaluated in vitro for their antiarrhythmic activity and influence on some cardiovascular parameters. 

The results of these assays are collected in Table 1 and Table 2.

While quinidine and lidocaine clearly increased the threshold of ac-arrhythmia, procainamide and amiodarone showed weak activity (11% at 50 µM and 10% at 100 µM, respectively).

Concerning the investigated compounds, antiarrhythmic activity was found, once again, in all subsets (a)–(c) of quinolizidine derivatives, despite the large structural diversity of the moieties to which the quinolizidine ring is joined. Thus, the claimed link [4,5] between this structural feature (as truncated sparteine) and antiarrhythmic activity is further strengthened.

Indeed, with the exception of compounds **2** and **12**, the tested quinolizidine derivatives showed rather high activity, resulting in their being comparably or more active than lidocaine and procainamide. In some cases (**1** and **4**–**6**), the activity and potency of quinidine (the best reference drug) were reached and even largely exceeded. Compounds **4**, **1** and **6** (in order) were equipotent or up to 15-fold more potent than quinidine, while an outstanding potency (EC_50_ = 0.017 µM) was observed for compound **5**, which was 600 times more potent than quinidine and 8.8 times more potent than our previously described [5] most potent quinolizidine derivative **C,** as shown in Figure 2.

Compound **5** is the (one-carbon)-homolog of the previously described [5] N-(3,4,5-trimethoxybenzoyl)aminolupinane, which exhibited a lower antiarrhythmic potency (EC_50_ = 2.62 µM), but was still higher than that of the reference drugs. The elongation of the linker between the quinolizidine ring and the aromatic nucleus produced a 150-fold increase in potency.

A favorable effect of the linker length on the antiarrhythmic potency was previousy observed (compare compound **D** with the N-homolupinanoyl-2,6-dimethylaniline [4], and compound **F** with **E**), but the opposite effect was also observed in the present study by comparing the EC_50_ of compound **1** with **2**, as well as **6** with **8** (Table 1).

The outstanding potency of compounds **5**, in addition to (or instead of) an improved fitting and efficacy on the target, might be related to the presence in the molecule of two distinct pharmacophoric substructures (such as the quinolizidine ring and the 3,4,5-trimethoxy benzoyl moiety), with each being suitable to hit a different target to initiate multiple mechanisms of action. An appropriate distance between the pharmacophoric groups may be required to avoid or reduce any reciprocal hindrance to the respective target activation.

Indeed, some intrinsic anti-arrhythmic properties should be attributed to the trimethoxybenzoyl residue. Based on the known activities of reserpine (including the antiarrhythmic one [30]), several basic esters and amides of the 3,4,5-trimethoxybenzoic acid were investigated and found to be endowed with different degrees of various cardiovascular activities. Particularly, the 5-(3,4,5-trimethoxy)benzamido-2-methyl-trans-decahydroisoquinoline (M32) displayed an anti-arrhythmic activity (i.p. in mice) that was fivefold superior to that of quinidine [31,32,33].

Comparing the structures of **5** and **M32**, it is observed that the common aromatic moiety is linked to the respective basic nitrogen through a chain with the same number (6) of atoms, and that the two bicyclic systems (quinolizidine and decahydroisoquinoline) may adequately (even if not completely) overlap each other. Therefore, the two compounds could hit the same molecular target(s).

**Figure 4:**
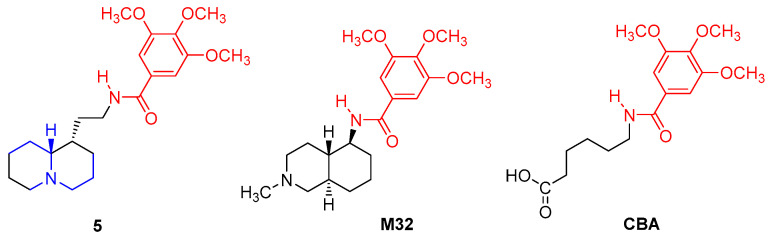
Comparison of the structures of compounds **5**, **M32**, and **CBA**.

However, non-basic derivatives of trimethoxybenzoic acid may also display valuable antiarrhythmic activity: in capobenic acid (**CBA**), where the trimethoxybenzoic acid is linked to ε-aminocaproic acid, the typical anti-fibrinolytic action of the latter is abolished, but a potent antiarrhythmic activity emerges [34] (Figure 4).

In contrast to the observed positive effect on antiarrhythmic activity, the lengthening of the linker produced the opposite effect on antihypertensive action: actually, compound **5** was found to be endowed with negligeable activity in spontaneously hypertensive rats [35], while its lower homolog displayed potent and long-lasting hypotensive activity in normal rabbits [36].

Differently from **5**, the compounds **1**–**4** of subset (a) are analogs of “orthopramides”. A class of D2 and 5-HT3 receptor antagonists, and/or 5-HT4 receptor agonists was endowed with variable degrees of antipsychotic, antiemetic or gastro-entero-prokinetic activities (sulpiride, metoclopramide, cisapride, etc.).

Compound **1**, still displaying moderate antiarrhythmic activity (EC_50_ = 3.66 µM), is a structural analog of sulpiride, whose (1-ethylpyrrolidin-2-yl)methyl moiety is replaced with the lupinyl ((quinolizidine-1-yl)methyl) residue.

Sulpiride displays a potent and selective antagonism compared to the D2 receptor, to which its psycholeptic, antiemetic and gastro-entero prokinetic activities are related, but shows neither antiarrhythmic nor arrhythmogenic potentials (as cisapride does) and exhibits a very low chance of prolonging the QT interval in ECG (IC_50_ for hERG blockade > 100 µM) [37,38].

Compared to sulpiride, compound **1** displays a 1000-fold lower affinity to the D2 receptor [3], and is also devoid [39] of affinity to the 5-HT4 receptor, which is determinative for the prokinetic activity of cisapride and analogs; nevertheless, compound **1** has appreciable effect on the intestine transit rate in mice, with a still undefined mechanism (which increases the length of the small intestine colored by charcoal by 8.8% versus 14% for sulpiride and 6.7% for metoclopramide [3]).

The N-lupinylbenzamido derivative **4**, which is endowed with an antiarrhythmic activity potency (EC_50_ = 10.67 µM) comparable to that of quinidine, is structurally related to the 6-methoxysalicylamides, which are described as particularly potent antagonists of the D2 receptor [40,41]. As observed for compound **1**, in this case, the introduction of a lupinyl in place of the (1-ethyl-pyrrolidin-2-yl methyl moiety reduced the affinity to the D2 receptor, although to a minor extent, due to the presence of the free hydroxy group [40,41]. However, the absence of any causal relation between antiarrhythmic activity and affinity to the D2 receptor was still observed.

Among the 2-(benzotriazol-2-yl)methyl benzimidazoles (subset b), compound **6** displays potent antiarrhythmic activity, with an EC_50_ = 0.68 µM, confirming the previously observed activity [27] against ouabain-induced arrhythmia in dogs. Structural modifications to compound **6**, such as epimerization (**7**) or elongation (**8**) of the basic side chain, decreased both activity and potency; the introduction of a substituent in position **5** of the benzimidazole ring was particularly deleterious (**9** and **10**).

Finally, the cytisine derivatives (**11** and **13**) displayed a moderate antiarrhythmic activity approaching that of lidocaine, while **14** was moderately active even at 1 µM concentration. The last compound, at a dose of 30 mg/Kg (*p. os* and *i.p.*) did not exhibit any sign of toxicity in mice and produced a 50% reduction in stress-induced ulcers in rats [29], making it deserving of further consideration. 

It is worth noting that N-(hydroxyethyl)cytisine (**11**) was already found by Russian authors to be active against aconitine-induced arrhythmia in anesthetized rats, with a potency close to that of lappaconitine (allapinine) [42,43,44].

The same Russian authors found that antiarrhythmic activity was also displayed by the N-hydroxyethyl-9,11-dibromocytisine [44] and by the N-[(2-hydroxy-2-phenyl)ethyl]cytisine [43]. However, in our hands [29], the latter compound did not display any inotropic or chronotropic effects on isolated left and right gp atria, respectively. To settle this issue, further investigation of cytisine derivatives would be worthwhile.

To better evaluate the pharmacological profile of the investigated quinolizidine derivatives **1**–**14**, it was deemed useful to compare their influence on additional cardiovascular parameters with that elicited by the reference drugs (Table 2).

With one exception, all compounds strongly decreased the developed tension on the driven gp left atria with EC_50_ in the range 0.009–0.083 µM, and were thus comparable to lidocaine (EC_50_ = 0.017 µM). Compound **11**, the least potent negative inotropic agent, displayed an EC_50_ = 0.14 µM, which is still 24 times lower than that of quinidine.

Anyway, this generally high negative inotropic activity does not invalidate the importance of several compounds as antiarrhythmic agents, particularly compound **5**, whose EC_50_ for the increase in the threshold of ac-arrhythmia remains lower than that of negative inotropism (0.017 µM versus 0.050 µM, respectively).

In preliminary investigation [29], it was observed that compound **14** (N-phenethylcytisine) at concentrations above 1 µM displayed a positive inotropism that reached its maximum at 31 µM, with a 48% increase in force, which was comparable to that exerted by trequinsin (an ultra-potent PDE inhibitor) at a 25 µM concentration [29].

Positive inotropism is rather unusual among antiarrhythmic agents, and it is worth noting that this dual activity is shared by matrine, another quinolizidine alkaloid, as illustrated by Zhou and Shan [45].

At concentrations above 100 µM, a negative inotropism was observed again. This alternating effect on the developed tension was not observed for the other cytisine derivatives.

Concerning the chronotropic activity (Table 2) detected on the spontaneously beating right atria, amiodarone and quinidine (at 100 and 50 µM, respectively) exerted a strong negative effect (72–86%), while procainamide and lidocaine showed a moderately positive effect (9% and 29%, at 100 µM and 5 µM, respectively).

All, but one (**3**) tested compound displayed negative chronotropic activity, which was, generally, rather modest; thus, it was only possible to calculate the EC_50_ values for three compounds in subset (b) (**6**, **7** and **8**). For compound **6**, the negative chronotropic activity was comparable (EC_50_ = 11.15 µM) to that of amiodarone (EC_50_ = 14.95 µM), but for the corresponding homolog **8,** a remarkable (507-fold) increase in negative activity was observed (EC_50_ = 0.019 µM).

The vasorelaxant activity, represented by the inhibition of the calcium-induced contraction of K^+^-depolarized (80 µM) gp aortic strips, was also quite modest. Compounds **1**, **2**, **3** and **11** were practically inactive, as were amiodarone and procainamide. All other compounds inhibited aortic strip contractions of 11–36% at 50–100 µM, as did lidocaine and quinidine.

Finally, the present work further supports the idea that the quinolizidine ring serves as the pharmacophore for antiarrhythmic activity, linked to largely diversified (as nature and dimension) aromatic moieties. 

Among these, the ones bearing an *arylcarbamido* group (**A** and **B** of Figure 2; **1** and **5** of Figure 3) appear to be more promising in the generation of valuable leads for antiarrhythmic drugs.

However, it should not be excluded that some particularly aromatic moieties could act as independent pharmacophores, in addition to the quinolizidinic one. This may be the case with the trimethoxybenzoyl residue of compound **5**.

Therefore, further investigations will be carried out by synthesizing novel arylcarbamido quinolizidines (widening subset (a)), but also by synthesizing novel 3,4,5-trimethoxybenzoyl derivatives devoid of the quinolizidine nucleus but containing differently truncated sparteine moieties.

Additionally, the observed antiarrhythmic and inotropic activities (at particular concentrations) for the cytisine derivatives **14** suggest **that** the investigation could be extended to other cytisine derivatives in order to achieve dual synchronically acting agents. 

## 3. Materials and Methods

### 3.1. Chemistry 

All 14 investigated compounds (Figure 3) were previously described: **1** and **3** [3]; **2** and **4** [39]; **5** [35]; **6** [26]; **7** and **8** [28]; **9** and **10** [27]; **11**–**14** [29].

**Scheme 1:**
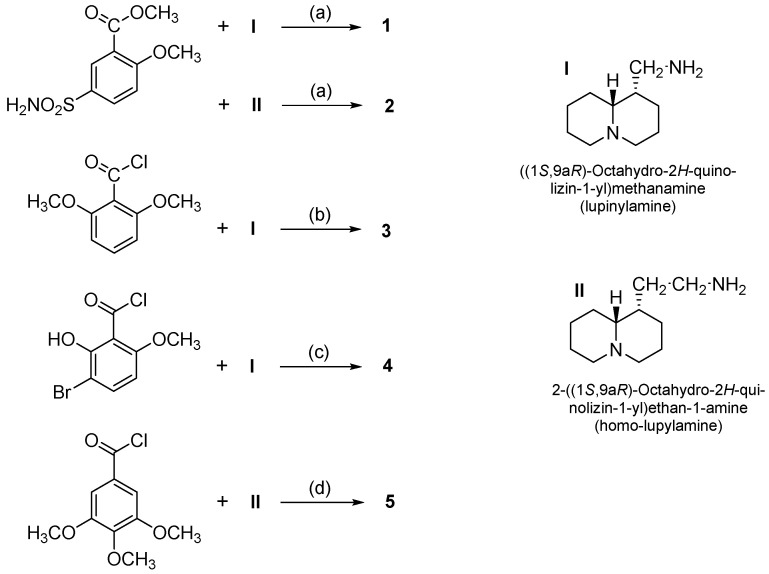
Reagents and conditions: (a) MeOH, 110 °C, 2 h (pressure tube); (b) Toluene, 80 °C, 3 h; (c) CH_2_Cl_2_, room temperature, 20 h; (d) benzene, TEA, reflux, 3 h.

**Scheme 2:**
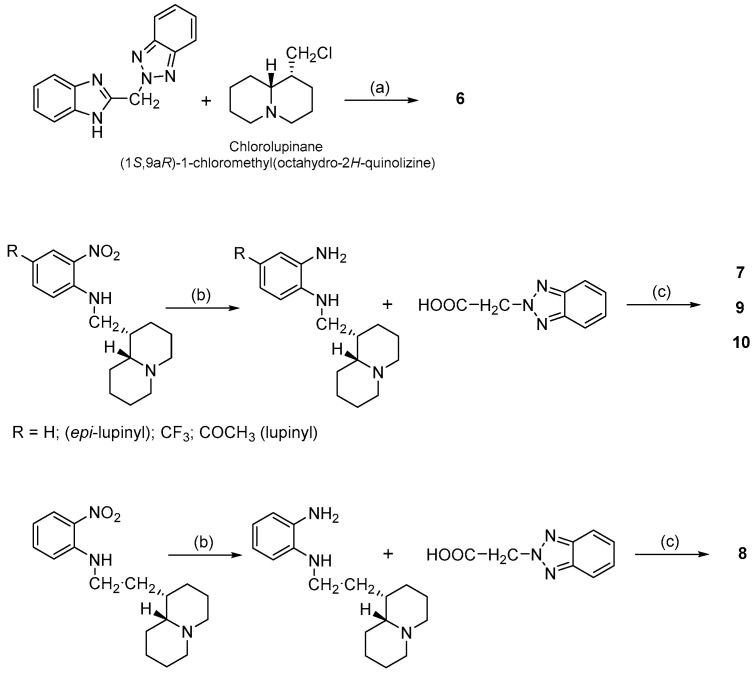
Reagents and conditions: (a) DMF, NaNH_2_, 140–145 °C; (b) H_2_, Pd/C, room temperature, atmosphere pressure; (c) 2 moles of acid per mole of amino compounds; 170–180 °C, 2 h.

**Scheme 3:**
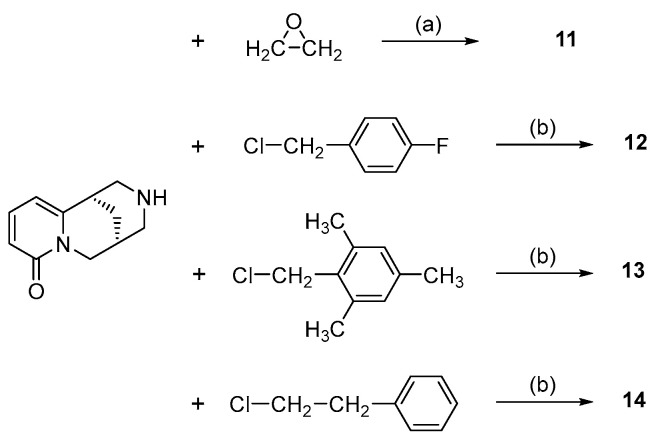
Reagents and conditions: (a) CHCl3, 45 °C, 4 h (pressure tube); (b) acetone, reflux, 1–2 h, 2 moles of cytisine per one of the chloro compounds.

The synthetic sequences are summarized in Scheme 1, Scheme 2 and Scheme 3.

The purity of compounds **1**–**14** was checked through m.p., TLC, and elemental analysis, and occasionally a few compounds were chromatographed on alumina (dry ether or dichlorometane as solvents) to restore high purity.

### 3.2. “In Vitro” Activity

Guinea pigs of both sexes (200–400 g), obtained from Charles River (Calco, Como, Italy), were used. Animals were housed in accordance with the ECC Council Directive on the protection of animals used for experimental and other scientific purposes (Directive 2010/63/EU of the European Parliament and of the Council) and the WMA Statement on the use of animals in biomedical research. All procedures followed the guidelines of the Animal Care and Use Committee of the University of Bologna (Bologna, Italy). Ethics Committee approval was reported and numbered “Protocol PR 21.79.14” from the Committee for Animal Research Protocols (Comitato Etico Scientifico for Animal Research Protocols) in accordance with D.L. vo 116/92. Guinea pigs were sacrificed by cervical dislocation.

Compounds **1**–**14** were tested for anti-arrhythmic activity in isolated guinea pig myocardium: the left atria were driven at 1 Hz and the right atrium beat was driven spontaneously to assess inotropic and chronotropic effects, respectively. Finally, K^+^-depolarized guinea pig aortic strips were used to evaluate calcium antagonist activity (as an expression of vasorelaxant activity). In all cases, compounds were added cumulatively.

Specifically, anti-arrhythmic activity was assessed by applying a sinusoidal alternating current (50 Hz) of increasing intensity to the isolated left atria driven at 1 Hz to induce arrhythmias and assessing the “ac arrhythmia threshold” (the current intensity at which an extra beat occurs) before and after the addition of the compound to the tissue bath.

Since ac-current-induced arrhythmias are mainly caused by increased Na^+^ conductance in cardiomyocytes, this method is particularly suitable for studying anti-arrhythmic drugs acting as Na^+^ channel inhibitors (Class I, including Sparteine) [46]. In any case, this model avoids the damage, toxicity, and drug–drug interactions caused by other chemical methods used to induce arrhythmias [47].

#### 3.2.1. Heart Preparation

After thoracotomy, the heart was immediately removed and cleaned. The left and right atria were isolated from the ventricles and separately prepared, as previously described, to test antiarrhythmic, inotropy and chronotropy activities [48].

#### 3.2.2. Aorta Preparation

The thoracic aorta was removed, placed in Tyrode solution, cleaned, and prepared as previously described [49].

### 3.3. Statistical Analysis

Data were analyzed using the Student’s test and presented as mean (M) ± SEM [50]. Significance (*p* < 0.05) was detected between the control and experimental values at each concentration. EC_50_ values were calculated for compounds that increased the ac arrhythmia threshold by more than 50%. Drug potencies, defined as EC_50_, were calculated from cumulative log concentration–response curves (probit analysis according to Litchfield and Wilcoxon [50] or using GraphPad Prism software [51,52] with appropriate pharmacological preparations.

## 4. Conclusions

Fourteen lupinyl-, homolupinyl-, and cytisine-derived compounds, all containing the quinolizidine ring in their structures, were assayed for antiarrhythmic activity and other cardiovascular effects on isolated gp heart tissues in comparison to well-established reference drugs.

The tested compounds were grouped into three subsets according to the different kind of aromatic fragments that are grafted to the quinolizidine nucleus: antiarrhythmic activity was displayed by members of all subsets.

All compounds but two compared favorably with the reference drugs. Potent antiarrhythmic activity was observed for compounds (in order of increasing potency) **4**, **1**, **6**, and **5**; the last of which was very outstanding, with an EC_50_ = 17 nM. It is suggested that the unusual potency of compound **5** might be related to the presence of two distinct pharmacophoric substructures in the molecule (the quinolizidine ring and the trimethoxybenzoyl group), each able to hit different targets to produce convergent antiarrhythmic effects.

Compound **5**, with its high potency and favorable cardiovascular profile, represents an interesting lead, and is deserving of ADME studies with the aim of eventually producing an antiarrythmic drug. Also appreciable is the benzimidazole derivative **6**, with submicromolar potency (EC_50_ = 0.68 µM) for antiarrhythmic activity, while the negative chronotropism (EC_50_ = 6.36 µM), was less than that of amiodarone (EC_50_ = 5.57 µM).

Compound **1**, whose valuable cardiovascular profile is associated with gastro-enteric prokinetic activity, is worth noting, as this might be profitable in the case of concomitant (and reciprocally worsening) cardiac and gastric pathologies [53].

The cytisine derivative **14**, even if less potent than the foregoing compounds, still increased the threshold of ac-arrhythmia by 16% at 1 µM concentration, at which point it started to display an unusual positive inotropic activity, which could be very useful in arrhythmia associated with heart failure.

In conclusion, the above-mentioned compounds deserve further investigation, and future research will mainly focus on two directions:

(1) The definition of a more complete pharmacological profile and of the mechanisms of action of the relevant compounds;
(2) The synthesis of novel compounds bearing the arylcarbamido group and synthesis of novel cytisine derivatives to explore the possibility of achieving dual-acting (antiarrhythmic and positive inotropic) agents.

## Figures and Tables

**Table 1:** Anti-arrhythmic activity of compounds **1**–**14** (Figure 3).

Compd	Max% Increase in Threshold of Ac-Arrhythmia after Pretreatment with Compound *^a^* (M ± SEM)	EC_50_ *^b^* (μM)	95% Conf Lim (μM)
Amiodarone	10 ± 0.5 *^c^*		
Lidocaine	34 ± 2.6		
Procainamide	11 ± 0.4		
Quinidine	69 ± 0.4	10.26	8.44–12.46
*N-(Quinolizidinyl-alkyl)-benzamides-related compounds*			
**1**	59 ± 1.7	3.66	2.10–6.38
**2**	9 ± 0.6 *^d^*		
**3**	17 ± 0.4 *^e^*		
**4**	92 ± 1.3	10.67	7.56–14.91
**5**	104 ± 3.4	0.017	0.0068–0.046
*1-(Quinolizidinyl)alkyl-2-(benzotriazol-2-yl)methyl benzimidazoles-related compounds*			
**6**	75 ± 1.8 *^d^*	0.68	0.47–0.98
**7**	37 ± 3.4 *^f^*		
**8**	39 ± 1.7 *^d^*		
**9**	16 ± 0.9		
**10**	20 ± 0.4		
*N-Substituted cytisines-* *related compounds*			
**11**	26 ± 0.3 *^c^*		
**12**	4 ± 0.2		
**13**	27 ± 1.3 *^c^*		
**14**	16 ± 0.7 *^g^*		

*^a^* Increase in threshold of ac-arrhythmia: increase in current strength of 50 Hz alternating current required to produce arrhythmia in guinea pig left atria driven at 1 Hz in the presence of each tested compounds at 5 × 10^−^^5^ M (unless otherwise stated). For all data *p* < 0.05, with the exception of compound 12. *^b^* Calculated from log concentration–response curves (probit analysis according to Litchfield and Wilcoxon with *n* = 6–8). When maximum effect was <50%, the EC_50_ values were not calculated. *^c^* At 10^−^^4^ M. *^d^* At 10^−^^5^ M. *^e^* At 10^−^^7^ M. *^f^* At 5 × 10^−^^6^ M. *^g^* At 10^−^^6^ M.

**Table 2:** Influences of compounds **1**–**14** on cardiovascular parameters.

	Heart						Aorta
	Left Atria			Right Atria			
	Negative Inotropy			Negative Chronotropy			Vasorelaxant
Compd	IA *^a^* (M ± SEM)	EC_50_ *^b^* (μM)	95% conf lim (μM)	IA *^c^* (M ± SEM)	EC_50_ *^b^* (μM)	95% conf lim (μM)	IA *^d^* (M ± SEM)
Amiodarone	30 ± 2.6 *^e^*			72 ± 4.5 *^e^*	14.95	11.07–20.16	3 ± 0.1 *^g^*
Lidocaine	88 ± 3.0	0.017	0.012–0.024	29 ± 0.9 *^#^*^,*j*^			14 ± 0.9
Procainamide	92 ± 1.4 *^f^*	0.014	0.011–0.017	9 ± 0.6 *^#,e^*			3 ± 0.2
Quinidine	71 ± 3.6 *^g^*	3.38	2.69–4.25	86 ± 0.5	25.31	14.45–44.32	30 ± 1.6 *^g^*
*N-(Quinolizidinyl-alkyl)-benzamides-related compondds*							
**1**	92 ± 1.4 *^h^*	0.037	0.027–0.051	24 ± 1.3 *^k^*			5 ± 0.2
**2**	93 ± 1.4 *^i^*	0.0091	0.002–0.021	2 ± 0.1 *^h^*			3 ± 0.2
**3**	75 ± 2.3 *^i^*	0.011	0.0079–0.014	25 ± 1.6*^#^*			2 ± 0.1
**4**	98 ± 1.3	0.021	0.016–0.027	46 ± 2.2			36 ± 1.3
**5**	85 ± 2.2	0.050	0.035–0.071	25 ± 0.9 *^h^*			22 ± 1.6
*1-(Quinolizidinyl)alkyl-2-(benzotriazol-2-yl)methyl benzimidazoles-related compounds*							
**6**	91 ± 2.4 *^h^*	0.046	0.035–0.061	67 ± 0.7	11.15	9.05–13.74	25 ± 1.7 *^g^*
**7**	93 ± 2.7 *^j^*	0.083	0.064–0.11	69 ± 1.3 *^h^*	0.49	0.43–0.65	16 ± 1.1
**8**	92 ± 1.3	0.022	0.015–0.031	83 ± 2.4	0.019	0.014–0.026	19 ± 1.2
**9**	87 ± 1.1 *^j^*	0.056	0.042–0.076	44 ± 1.5 *^h^*			24 ± 1.6
**10**	94 ± 3.4	0.021	0.014–0.032	22 ± 1.2 *^e^*			11 ± 1.0
*N-Substituted cytisines-* *related compounds*							
**11**	86 ± 2.2 *^h^*	0.14	0.095–0.20	35 ± 1.4 *^k^*			0.3 ± 00.1
**12**	92 ± 1.8 *^h^*	0.018	0.013–0.026	26 ± 1.9			25 ± 1.4
**13**	87 ± 1.4 *^h^*	0.044	0.028–0.068	20 ± 0.3 *^h^*			32 ± 2.2
**14**	71 ± 0.7 *^i^*	0.016	0.0081–0.023	47 ± 1.1 *^e^*			20 ± 1.6

*^a^* Intrinsic Activity (IA): decrease in developed tension on isolated guinea-pig left atrium driven at 1 Hz at 10^−^^6^ M (unless otherwise stated), expressed as percent changes from the control (*n* = 4–6). For all data *p* < 0.05. *^b^* Calculated from log concentration–response curves (probit analysis according to Litchfield and Wilcoxon with *n* = 6–8). When the maximum effect was <50% the EC_50_ (inotropy), the EC_50_ (chronotropy) and IC_50_ (vasorelaxant) values were not calculated. *^c^* Intrinsic Activity (IA): decrease in atrial rate on guinea-pig spontaneously beating isolated right atria at 5 × 10^−^^5^ M (unless otherwise stated), expressed as percent changes from the control (*n* = 6–8). Pretreatment heart rate ranged from 170 to 195 beats/min. For all data *p* < 0.05, with the exception of compound **2**. *^d^* Intrinsic Activity (IA): percent inhibition of calcium-induced contraction on K^+^-depolarized guinea–pig aortic strips at 10^−^^4^ M (unless otherwise stated). The 10^−^^4^ M concentration gave the maximum effect for all but one (6, at 5 × 10^−^^5^ M) compounds. *p* < 0.05 for amiodarone, procainamide and compounds **1**, **2**, **3**, and **11**. *^e^* At 10^−^^4^ M. *^f^* At 5 × 10^−^^7^ M. *^g^* At 5 × 10^−^^5^ M. *^h^* At 10^−^^5^ M. *^i^* At 10^−^^7^ M. *^j^* At 5 × 10^−^^6^ M. ^#^ Positive chronotropic effect. *^k^* At 10^−^^6^ M.

## Data Availability

Not applicable.
